# Evaluating the prebiotic activity of arabinogalactan on the human gut microbiota using 16S rRNA gene sequencing and Raman-activated cell sorting

**DOI:** 10.20517/mrr.2025.29

**Published:** 2025-08-14

**Authors:** Hamid Rasoulimehrabani, Sanaz Khadem, Adnan Hodžić, Miriam Philipp, Rebecca Gallo, Georgi Nikolov, Joana Séneca, Julia Ramesmayer, Patrik Sivulič, David Berry

**Affiliations:** ^1^Center for Microbiology and Environmental Systems Science, Department of Microbiology and Ecosystem Science, University of Vienna, Vienna 1030, Austria.; ^2^Doctoral School in Microbiology and Environmental Science, University of Vienna, Vienna 1030, Austria.; ^3^Joint Microbiome Facility of the Medical University of Vienna and the University of Vienna, Vienna 1030, Austria.; ^4^Department of Natural Drugs, Faculty of Pharmacy, Masaryk University in Brno, Brno 612 00, Czech Republic.

**Keywords:** Arabinogalactan, *Bifidobacterium longum*, gut microbiota, prebiotics, Raman-activated cell sorting, microbial cross-feeding

## Abstract

**Background:** Arabinogalactan is a complex plant-derived polysaccharide proposed to function as a selective prebiotic, yet the microbial taxa directly involved in its metabolism and the cooperative dynamics within the gut microbiota remain incompletely defined.

**Methods:** Here, we combined community-level sequencing with targeted single-cell activity profiling to investigate how arabinogalactan shapes gut microbial composition and function. Fecal samples from ten healthy individuals were incubated *ex vivo* with arabinogalactan, and microbial responses were assessed using 16S rRNA gene amplicon sequencing alongside Raman-activated cell sorting (RACS) and coculture experiments.

**Results:** Arabinogalactan consistently enriched *Bifidobacterium* and *Gemmiger* across donors, with *Bifidobacterium* also responding to galactose and *Gemmiger* and *Blautia* stimulated by arabinose, the two monosaccharide components of arabinogalactan. RACS enabled the selective isolation of metabolically active arabinogalactan responders, including *Bifidobacterium longum* (*B. longum*) and *Faecalibacterium prausnitzii*, along with other strains from the phyla *Actinomycetota*, *Bacteroidota*, and *Bacillota*. Notably, coculture experiments revealed that *B. longum* not only degraded arabinogalactan efficiently but also supported the growth of non-degrading species via metabolic cross-feeding. These cooperative interactions highlight *B. longum* as a keystone species in arabinogalactan utilization and suggest broader community-level benefits from its activity.

**Conclusion:** Together, our findings demonstrate arabinogalactan’s bifidogenic effect and its potential to promote functionally important microbes within the gut ecosystem. This study also highlights the utility of RACS for linking microbial identity to function, enabling the targeted recovery of active strains from complex communities.

## INTRODUCTION

The human gut microbiota is essential for maintaining host health through its roles in nutrient metabolism, immune regulation, and protection against pathogens^[1]^. Microbial community composition in the gut is influenced by various factors, with diet recognized as a major determinant^[2]^. At baseline, the adult gut microbiota predominantly consists of bacteria belonging to the phyla *Bacillota*, *Bacteroidota*, *Actinomycetota*, *Verrucomicrobiota*, and *Pseudomonadota*^[3-5]^. However, the relative abundance of these phyla exhibits considerable variability among individuals, underscoring substantial inter-individual differences in microbiota structure and functionality^[6]^. Non-digestible dietary fibers are increasingly recognized for their ability to selectively modulate the composition and function of the gut microbiota^[7]^. These fibers, often termed prebiotics, resist digestion by human enzymes, reaching the large intestine intact, where they are selectively fermented by certain gut microbes, thereby conferring health benefits to the host^[8]^. Well-studied prebiotics include oligosaccharides such as fructooligosaccharides (FOS) and galactooligosaccharides (GOS), as well as polysaccharides like inulin, resistant starch, and beta-glucans, which have been shown to selectively stimulate the growth of beneficial gut microbes such as *Bifidobacterium* and *Lactobacillus*^[8-10]^.

Arabinogalactan is a plant-derived polysaccharide that has recently been proposed as a potential prebiotic^[11,12]^. Prebiotics are defined as substrates selectively utilized by host microorganisms that confer a health benefit^[13]^. This definition emphasizes both microbial selectivity and demonstrable benefit to the host. In this context, arabinogalactan fulfills key prebiotic criteria by reaching the colon undigested, being selectively fermented by beneficial gut bacteria such as *Bifidobacterium*, and contributing to the production of health-promoting short-chain fatty acids (SCFAs)^[14]^. It is naturally abundant in certain dietary sources, notably larch wood and legumes^[11,15]^. Arabinogalactan is a structurally complex, branched polysaccharide typically composed of a galactan backbone with β-1,3- and/or β-1,6-glycosidic linkages, and contains side chains made of arabinose and galactose residues^[16]^. Fermentation of arabinogalactan by the gut microbiota results in the production of SCFAs, particularly propionate and butyrate, along with other organic acids^[17,18]^. These metabolites can contribute to lowering intestinal pH and promoting a gut environment associated with improved host health^[19]^. Arabinogalactan supplementation has been shown to selectively increase the abundance of *Bifidobacterium* species, indicating its potential bifidogenic effect^[14,20,21]^. However, the specific microbial taxa directly involved in arabinogalactan metabolism, as well as the dynamics of their metabolic interactions within the broader microbial ecosystem, remain incompletely characterized^[22-25]^. Efficient utilization of complex polysaccharides like arabinogalactan can involve cooperative interactions among multiple microbial species^[24,26]^. Primary degraders can break down complex polysaccharides via CAZymes, releasing metabolites that support secondary consumers^[27]^. Identifying these microbial interactions is key to understanding how polysaccharides such as arabinogalactan selectively modulate gut microbiota and promote host health^[14,27]^. These microbial shifts can benefit the host by enhancing SCFA production, supporting gut barrier integrity, and modulating immune responses^[28]^.

To investigate the microbial response to arabinogalactan at both community and single-cell resolution, we employed an integrated *ex vivo* approach using fecal samples from ten healthy donors. First, we characterized community-level shifts through 16S rRNA gene amplicon sequencing, assessing compositional changes driven by arabinogalactan supplementation. Next, we utilized single-cell Raman microspectroscopy combined with deuterium (D_2_O) labeling to identify individual microbial cells actively metabolizing arabinogalactan. Metabolically active bacteria were selectively isolated via Raman-activated cell sorting (RACS) and subsequently characterized to confirm their physiological capacity to degrade arabinogalactan. Finally, coculture experiments between a primary arabinogalactan degrader - *Bifidobacterium longum* (*B. longum*) - and metabolically active, non-degrading isolates provided insights into potential cooperative interactions, illustrating how *B. longum* facilitates secondary growth via metabolic cross-feeding. Together, our results provide mechanistic insight into how arabinogalactan selectively enriches specific gut microbes and promotes cooperative interactions, supporting its role as a functionally selective prebiotic.

## METHODS

### Sample collection and incubation

Stool samples were collected from 10 healthy adults (five males and five females; mean BMI: 24.43 ± 3.69). Participants were excluded if they had taken antibiotics, consumed prebiotic or probiotic products in the past six months, or had a known history of gastrointestinal disorders or recent digestive illness^[29,30]^. Participants collected their own stool samples using sterile screw-cap containers equipped with built-in collection spoons (Sarstedt, Germany). The study was approved by the Ethics Committee of the University of Vienna, and all individuals provided written informed consent (reference number: 00161).

Immediately following collection, fecal material was transferred into an anaerobic chamber (gas mixture: 85% N_2_, 10% CO_2_, 5% H_2_) to maintain strict anaerobic conditions^[31,32]^. To assess microbiota responses, fecal homogenates were incubated with arabinogalactan, arabinose, and galactose (2 mg/mL final concentration, Carl Roth and Sigma-Aldrich)^[7]^, while unamended samples were used as negative controls. Samples were homogenized in 2× PBS by vigorous vortexing (2-3 min), passed through a 40 μm mesh filter (Corning, Germany) to remove large debris, and diluted 1:10 with 2× PBS.

Each sugar was dissolved in D_2_O and added to sterile Hungate tubes containing 2 mL of the fecal homogenate. The final volume was adjusted to 4 mL, yielding a 50% D_2_O concentration. Incubations were carried out anaerobically at 37 °C for 6 and 24 h. Following incubation, cells were rinsed with PBS to eliminate residual D_2_O. For downstream processing, 1 mL of each sample was fixed in 50% ethanol/PBS and stored at -20 °C, while an additional 1 mL aliquot was frozen at -80 °C for subsequent nucleic acid and metabolite analysis. For RACS, aliquots were preserved in 20% glycerol/PBS and stored at -80 °C in crimp-sealed vials.

The entire preparation process, including sample homogenization, filtration, and substrate addition, took approximately 30 min. This point was considered the initial time point (0 h) in the dataset and used as the reference for all downstream analyses.

### DNA extraction and 16S rRNA amplicon sequencing

To assess shifts in microbial composition, DNA was isolated from stool samples harvested at 0, 6, and 24 h after treatment with arabinogalactan, arabinose, galactose, or without any amendment. DNA was extracted using the QIAamp DNA Mini Kit (Qiagen, Germany), incorporating a lysozyme pre-treatment step to enhance cell lysis and optimize DNA recovery^[33]^. The 16S rRNA gene was amplified using a two-step PCR approach with dual barcoding, allowing for high-resolution profiling of microbial community composition^[34]^. Sequencing was performed at the Joint Microbiome Facility (JMF) of the Medical University of Vienna and the University of Vienna (Project ID: JMF-2307-05). The resulting sequence datasets have been deposited in the NCBI Short Read Archive under the BioProject accession number PRJNA1244259.

### Single-cell Raman analysis of arabinogalactan-stimulated gut microbiota

To assess microbial responses, fecal samples were exposed to arabinogalactan for 6 and 24 h, while parallel incubations without amendment were used as controls. To maintain cellular integrity, samples were fixed in a 1:1 mixture of ethanol and PBS. For Raman measurements, 1 μL of each fixed sample was applied to an aluminum-coated slide (Al136; EMF Corporation, USA) and allowed to air-dry at 30 °C^[35,36]^. Residual buffer components were removed by dipping the slides twice in ice-cold Milli-Q water (Millipore, Austria), followed by air drying^[37]^.

Spectral acquisition was performed using a confocal Raman microspectroscope (LabRAM HR800, Horiba Scientific, France) equipped with a 532 nm Nd:YAG laser and a diffraction grating of 300 grooves/mm^[38]^. Prior to measurement, the laser was calibrated using a silicon crystal standard, and the sample slide was brought into focus using a 100× objective^[39]^. Cell clusters were visually selected, and a mapping grid was applied to the designated area for targeted measurement.

An acquisition time of 0.3 s was chosen to match the conditions used in RACS, allowing direct comparison between the two approaches. Preliminary tests indicated that using 4 accumulations provided the clearest spectra while minimizing laser exposure and preserving cell integrity. A delay time of 0 s was used, and binning was set to 1 to achieve the highest spectral resolution.

A total of 30 to 40 single microbial cells were analyzed per sample. Spectra were considered valid if they showed a clear C–H stretch (2,800-3,100 cm^-1^) and a phenylalanine peak (~1,000 cm^-1^), confirming signal origin from cellular material^[40,41]^. Deuterium incorporation was identified via the C–D stretch (2,040-2,300 cm^-1^), reflecting active metabolism^[35]^.

The percentage of deuterium-labeled bonds (%CD) was calculated as the ratio of C–D to total C–H and C–D signals using the Scattr analysis tool (https://shiny.lisc.univie.ac.at/scattr/). The threshold for metabolic activity was determined based on %CD values from cells incubated without the addition of D_2_O (water control), calculating the mean plus 3 times the standard deviation (SD)^[38]^.

### Targeted isolation of arabinogalactan-responsive cells via RACS

RACS was performed on fecal samples incubated for 6 h with D_2_O and arabinogalactan to isolate metabolically active cells. After incubation, cells were preserved in 20% glycerol/PBS and stored at -80 °C. For sorting, frozen samples were thawed, pelleted at 9,000 × *g* for 7 min, and washed twice with 0.3 M PBS/glycerol solution to minimize osmotic stress^[42]^. The final cell pellet was resuspended in 500 μL of the same buffer and loaded into a 500 μL Hamilton syringe under anaerobic conditions. The RACS platform consisted of a confocal Raman microscope (532 nm, 90 mW), optical tweezers using a 1,064 nm Nd:YAG laser (500 mW), and a polydimethylsiloxane (PDMS)-based microfluidic sorting device^[42]^. The PDMS chips were fabricated by mixing base elastomer (Sylgard 184TM, Dow Corning, Michigan, USA) and curing agent at a 10:1 ratio, polymerized at 75 °C, and mounted on a glass coverslip^[42]^. During the sorting process, cells were sorted by their Raman spectral profiles, allowing deuterium-labeled cells to be directed to the collection outlet, whereas unlabeled cells were guided toward the waste outlet^[43]^. Cells were identified and sorted based on Raman spectra using automated MATLAB software (version 4.2) based on two indices. The cell index (Pc), which indicate cell capture, was defined as the integrated Raman signal between 1,620-1,670 cm^-1^ relative to the surrounding medium, and the labeling index (PL) was calculated as the ratio of C–D stretch (2,040-2,300 cm^-1^) to a reference region (1,850-1,900 cm^-1^)^[42]^. Thresholds for Pc and PL were set based on glucose-incubated controls without D_2_O (Pc = 1.1; PL = 6.0). Labeled cells were directed into a collection outlet using optical tweezers, while unlabeled cells were routed to waste.

The sorting process for each sample lasted approximately 60 to 90 min. After sorting, 50 μL of the collected cell suspension was immediately plated on YCFA agar plates and incubated anaerobically at 37 °C. A 50 μL aliquot of the sheath buffer was also plated as a negative control. Colonies recovered after initial plating were subsequently transferred onto YCFA-arabinogalactan plates for confirmation of growth, and successfully grown isolates were preserved as glycerol stocks at -80 °C. The RACS platform has an average throughput of up to 500 cells per hour and a sorting accuracy of 98.3% ± 1.7%^[43]^. For each donor, the total number of D_2_O-labeled cells analyzed, sorted, and successfully cultivated was recorded. The post-sorting cultivation success rate, defined as the percentage of colonies recovered relative to the number of labeled cells sorted, ranged from 0.6% to 32.96% across donors [Supplementary Table 1].

### 16S rRNA gene sequencing of RACS-isolated bacteria

To genetically characterize the bacterial isolates obtained from RACS, colony PCR targeting the 16S rRNA gene was conducted. All PCR mixtures were assembled in a sterile PCR hood to prevent contamination. The reaction mix (50 μL total volume) contained the following components: 5 μL of 10× Green Dream Taq Buffer with 20 mM MgCl_2_ (Thermo Fisher Scientific, USA), 5 μL of 2 mM dNTP mix (Thermo Fisher Scientific), 1 μL of each primer, 616V (5′-AGA GTT TGA TYM TGG CTC AG-3′) and 1492R (5′-GGT TAC CTT GTT ACG ACT T-3′) with the final concentration of 50 μM (Thermo Fisher Scientific), 0.5 μL of 20 mg/mL BSA (Thermo Fisher Scientific), 0.5 μL of DreamTaq DNA Polymerase (5 U/μL) (Thermo Fisher Scientific), and 37 μL of nuclease-free water (Thermo Fisher Scientific). PCR amplification conditions were as follows: initial denaturation at 95 °C for 3 min; 30 cycles of 95 °C for 30 s, 56 °C for 30 s, and 72 °C for 90 s; followed by a final extension at 72 °C for 10 min. Amplified fragments were checked by electrophoresis on 1% agarose gels and purified using the InnuPREP PCRpure Kit (Analytik Jena, Germany) as per the manufacturer’s instructions. DNA concentrations were quantified using a Nanodrop spectrophotometer (Thermo Fisher Scientific, USA). The purified PCR products were submitted to Microsynth Austria GmbH (Vienna, Austria) for Sanger sequencing. Resulting chromatograms were processed and evaluated using 4Peaks (version 1.8, Nucleobytes, Amsterdam) and aligned in Serial Cloner (version 2.6). Potential chimeric sequences were identified using the online DECIPHER tool^[44]^. Taxonomic assignments were made by comparing the sequences against the NCBI nucleotide collection database (https://blast.ncbi.nlm.nih.gov/) with a minimum similarity threshold of 96%. Verified sequences were subsequently uploaded to GenBank under accession numbers PQ407681 - PQ407778.

### Growth curve analysis

Bacterial strains identified by Sanger sequencing were grown anaerobically at 37 °C in both solid and liquid YCFA-glucose (YCFA-G) medium. Once cultures reached the early stationary phase, as determined by OD600, cells were harvested, washed, and resuspended in fresh YCFA medium containing either arabinogalactan (YCFA-AG) or no supplement (YCFA-NA) as a control. Each culture was distributed into a sterile 96-well microplate (Costar 3595, Corning, NY, USA) in triplicate. The plate was incubated for 48 h at 37 °C using an anaerobic microplate reader (Multiskan^TM^ GO, Thermo Fisher Scientific), with optical density at 600 nm measured every 30 min and continuous shaking (5 s before each readout) controlled by SkanIt Software RE (v6.1.0.51). Growth kinetics were analyzed using R (https://www.r-project.org/), and the resulting culture supernatants were harvested and stored at -80 °C.

### Coculture evaluation

To investigate potential metabolic interactions, coculture experiments were performed using *B. longum* and eight non-degrading strains previously identified via RACS as unable to utilize arabinogalactan independently. These strains included *E. lenta*, *C. aerofaciens*, *P. coprocola*, *D. welbionis*, *R. bicirculans*, *P. faecium*, *P. merdae*, and *A. shahii*. Each strain was incubated anaerobically at 37 °C in YCFA medium supplemented with arabinogalactan (YCFA-AG), both as a monoculture and in coculture with *B. longum*. For coculture setups, equal volumes (50 µL) of log-phase cultures of *B. longum* and the respective non-degrader were mixed. Samples were collected at 0 and 24 h to monitor growth progression and capture late log-phase activity.

Strain-specific primers were designed using Primer3Plus to enable quantitative PCR (qPCR) analysis of strain abundance in both monocultures and cocultures^[45]^. The primers used for each strain were as follows: BifL-F (GAGATACGGCTTCCCTTCGG) and BifL-R (CATAATCCGCTGGCAACACG) for *B. longum* (Product length: 126 bp, Tm: 60 °C), Egg-F (CGCGGCCCATTAGGTAGTAG) and Egg-R (AGTCTGGGCCGTATCTCAGT) for *E. lenta* (Product length: 106 bp, Tm: 60 °C), Coll-F (TGCTACAATGGCCGGTACAG) and Coll-R (AGCAACTCCGACTTCATGGG) for *C. aerofaciens* (Product length: 114 bp, Tm: 60 °C), Phoc-F (CGTGAGGTGTCGGCTTAAGT) and Phoc-R (TCCTCGCATCTTACGATGGC) for *P. coprocola* (Product length: 105 bp, Tm: 60 °C), Dyso-F (GAGCTCGCGTCTGATTAGCT) and Dyso-R (TGTCTCAGTCCCAATGTGGC) for *D. welbionis* (Product length: 100 bp, Tm: 60 °C), Rumino-F (GAGCTCGCGTCTGATTAGCT) and Rumino-R (TGTCTCAGTCCCAATGTGGC) for *R. bicirculans* (Product length: 100 bp, Tm: 60 °C), Phas-F (AGTAAACGAGGAAGCCACGG) and Phas-R (AAGCCGCCTACATGCTCTTT) for *P. faecium* (Product length: 105 bp, Tm: 60 °C), Para-F (GTGTGTTTGAGGTAGGCGGA) and Para-R (GTAAGCTGCCTTCGCAATCG) for *P. merdae* (Product length: 84 bp, Tm: 60 °C), Alis-F (AGCTGGTTGGTGAGGTAACG) and Alis-R (TGGTCCGTGTCTCAGTACCA) for *A. shahii* (Product length: 89 bp, Tm: 60 °C). Primer specificity was validated using PCR with both specific and non-specific templates and optimized using gradient PCR to determine optimal annealing temperatures. For qPCR quantification, DNA standards were prepared by growing each strain to OD_600_ = 0.1, followed by DNA extraction. The 10-fold serial dilutions of the extracted DNA were used to establish standard curves for absolute quantification. The qPCR reactions were run in triplicate for each sample, enabling accurate quantification of bacterial abundance in both monocultures and cocultures across the selected time points. This setup allowed us to evaluate strain-specific growth responses and potential cross-feeding interactions mediated by *B. longum* during arabinogalactan degradation.

### Statistical analysis and reproducibility

All analyses were conducted in R statistical software (v4.2.1; https://www.r-project.org/)^[46]^. Data preprocessing and transformation were performed using the data.table, dplyr, and tidyr packages, and visualizations were generated with ggplot2 (v3.5.1)^[47]^. Microbial community-level differences were assessed using the vegan package (v2.6.4)^[48]^, applying Bray–Curtis dissimilarity and permutational multivariate analysis of variance (PERMANOVA)^[48]^. To evaluate taxon-level responses to dietary amendments, enrichment factors (EFs) were calculated by comparing genus-level relative abundances at 6 and 24 h to their corresponding baseline [no amendment (NA)] at the same time points. The EF was computed using the formula EF = 2A/(A + B) - 1, where A and B represent the relative abundances in treated and control conditions, respectively. This approach enabled genus-level comparisons across donors while keeping the output values within a defined and symmetric range between -1 and 1. Because we did not include external spike-ins or perform absolute quantification, EFs were calculated based on relative changes within each individual donor, comparing treated and control conditions separately for each donor. Genera with EF > 0 were considered enriched, and those with EF < 0 depleted. Statistical significance was assessed using z-scores derived from RA comparisons, with *P*-values adjusted using the Benjamini-Hochberg false discovery rate (FDR) method^[49]^. Genera with EF > 0 and FDR-adjusted *P* < 0.05 were considered significantly enriched. Bubble plots were used to visualize results, where bubble size reflected the relative abundance of baseline at the initial time point (0 h), bubble color encoded EF, and statistically significant genera were annotated with asterisks. DESeq2 (v1.38.3) was also used for differential abundance testing, modeling genus-level count data with a negative binomial distribution^[50]^. Donor identity and time point were included as variables in the model design. We selected DESeq2 for this analysis because it supports modeling donor identity and treatment effects, which was essential for accounting for the high inter-individual variability observed across donors. Genera with FDR-adjusted *P* < 0.05 were considered significantly differentially abundant. The combination of EF and DESeq2 provided complementary insights into both donor-specific and consistent taxon-level responses to arabinogalactan supplementation. To assess shifts in microbial community structure between arabinogalactan-amended and control samples, PERMANOVA was performed using the vegan package (v2.6.4) in R. The adonis function was applied to test for differences in community composition based on Bray–Curtis dissimilarity metrics computed from genus-level relative abundance profiles^[48]^. This multivariate approach allowed us to evaluate whether overall microbial community structures varied significantly between treatments, beyond changes at the individual taxon level.

## RESULTS

### Selective enrichment of gut microbes in response to arabinogalactan supplementation

**Figure 1:**
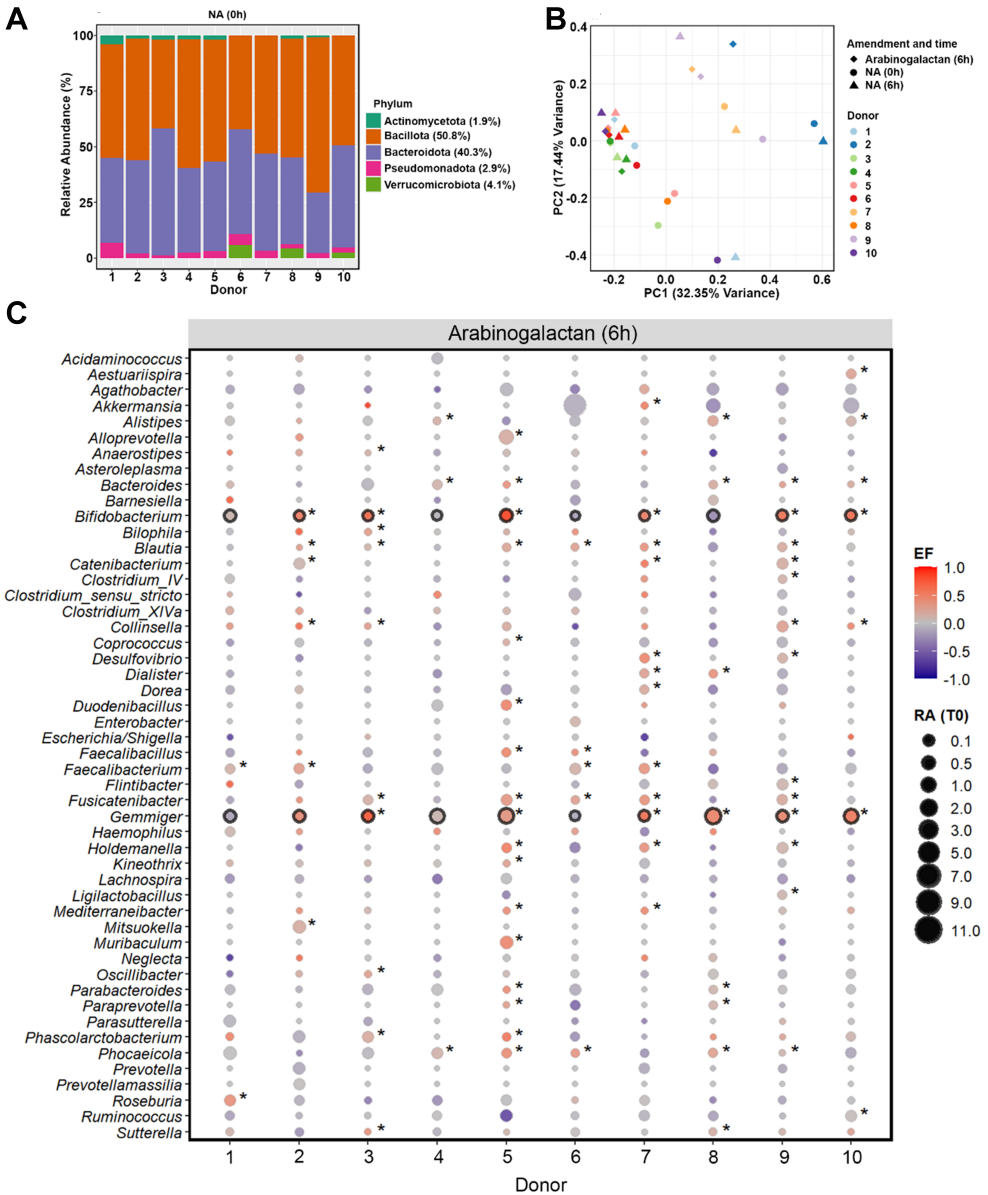
Baseline gut microbiota composition and response to arabinogalactan. (A) Relative abundances (%) of fecal microbiota at the phylum level across ten donors at the baseline (0 h). Average values for each phylum across all donors are provided in the legend; (B) PCoA of genus-level microbial communities, illustrating the clustering pattern of samples after 6-hour incubation with arabinogalactan, compared to NA samples at 0 and 6 h. Color and shape indicate donor identity and treatment condition, highlighting both individual and treatment-related differences; (C) Enrichment patterns of dominant bacterial genera after 6 h incubation with arabinogalactan. Bubble size indicates the relative abundance at 0 h, and color indicates the scaled EF, as described in the METHODS section. Genera with significant enrichment in individual donors are indicated by an asterisk, while those consistently enriched across all donors are highlighted with a black outline (DESeq2, Wald Test *P*.adj < 0.05, *n* = 30 samples). PCoA: Principal coordinate analysis; NA: no amendment; EF: enrichment factor.

To characterize the baseline microbial community structure prior to incubation, we performed 16S rRNA gene amplicon sequencing on fecal samples collected from all ten donors at the initial time point (0 h). Across the donors, the gut microbiota was predominantly composed of members of the phyla *Bacillota* (50.8%) and *Bacteroidota* (40.3%), with lower contributions from *Verrucomicrobiota* (4.1%), *Pseudomonadota* (2.9%), and *Actinomycetota* (1.9%) [Figure 1A].

Analysis of genus-level microbiome profiles revealed that most of the variation in microbial composition was explained by donor-specific differences, with inter-individual effects accounting for 71.3% of the total variation (*R*^2^ = 0.713, *P* = 0.001; *n* = 20 samples). This donor effect was also the main factor driving the clustering pattern observed in the principal coordinates analysis, indicating pronounced baseline differences in gut microbiota across individuals [Figure 1B]. Despite this variation, microbial communities incubated with arabinogalactan for 6 h showed a consistent shift relative to 0 and 6 h NA samples. A significant effect of arabinogalactan treatment on microbial community composition was detected (*R*^2^ = 0.121, *P* = 0.004), indicating that the induced shifts were consistently reproducible across distinct microbiota backgrounds.

To assess the extended short-term effects of supplementation, we incubated samples for 24 h. Similar to the 6 h time point, community composition remained strongly donor-driven (*R*^2^ = 0.703, *P* = 0.001), yet arabinogalactan treatment still explained a significant portion of the variation between groups (*R*^2^ = 0.123, *P* = 0.004). These results confirm that the observed community shifts are sustained over time and continue to reflect a robust and reproducible response to arabinogalactan across different individuals [Supplementary Figure 1].

We next identified bacterial genera that were selectively enriched in response to arabinogalactan after 6 h of incubation. Genus-level differential abundance analysis revealed that *Bifidobacterium* and *Gemmiger* showed a consistent and significant increase across the donors, but not in the NA samples (DESeq2, Wald Test *P.adj* < 0.05; Figure 1C, Supplementary Figure 2). This highlights a conserved response to arabinogalactan supplementation, suggesting these genera may play a central role in its metabolism. The enrichment of *Bifidobacterium* across the donor samples reflects a robust bifidogenic response to arabinogalactan treatment. This effect was not observed in unamended controls, highlighting that the enrichment was specifically induced by arabinogalactan. In addition, other taxa - including *Alistipes*, *Bacteroides*, *Blautia*, *Catenibacterium*, *Collinsella*, *Faecalibacterium*, *Oscillibacter*, *Parabacteroides*, *Phascolarctobacterium*, *Phocaeicola*, and *Ruminococcus* - showed significant enrichment in one or more donors, reflecting donor-specific shifts in microbial composition [Supplementary Data 1].

To assess whether these effects were maintained or intensified over time, we performed an additional incubation with arabinogalactan for 24 h. The enrichment patterns observed at 6 h were largely preserved, with *Bifidobacterium* and *Gemmiger* continuing to show significant increases across the donors [Supplementary Figure 3 and Supplementary Data 2]. In contrast, incubations NA for 6 or 24 h did not result in any consistent or significant enrichment at the genus level across donors [Supplementary Figure 2], supporting that the observed microbial changes were specifically driven by arabinogalactan treatment.

To further distinguish the effects of arabinogalactan from those of its constituent monosaccharides, we incubated the same donor samples with either galactose or arabinose for 6 and 24 h. Galactose consistently stimulated a significant enrichment of *Bifidobacterium* across the donors at both time points [Supplementary Data 3 and 4]. In contrast, arabinose triggered significant enrichment of *Blautia* at both time points, while *Gemmiger* was enriched at 6 h but not at 24 h. Notably, *Anaerostipes* showed significant enrichment across the donors specifically at 24 h [Supplementary Figures 4 and 5, Supplementary Data 5 and 6].

### Targeted cultivation of arabinogalactan-responsive bacteria using Raman-based activity profiling

**Figure 2:**
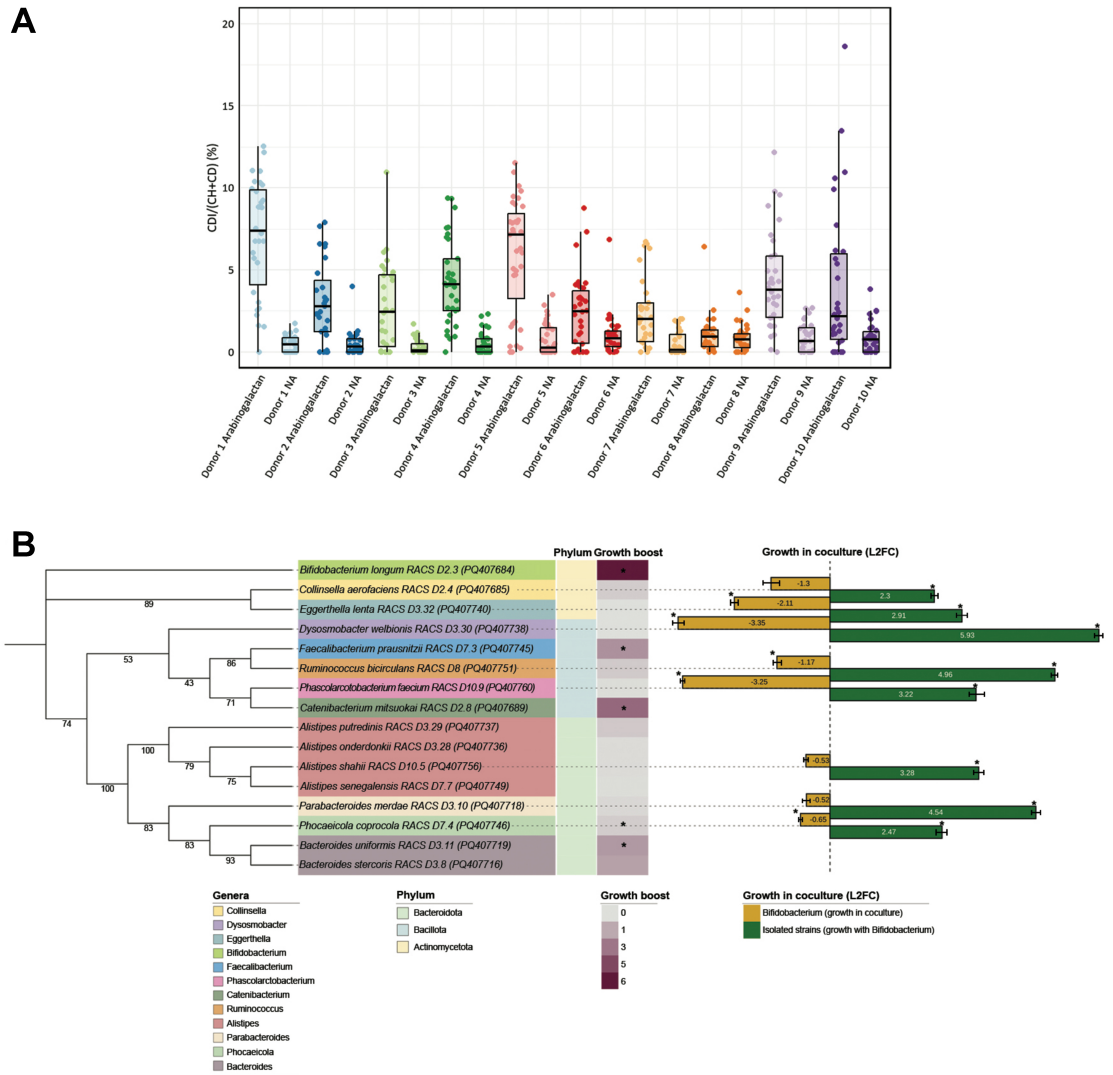
Raman-based detection and physiological analysis of arabinogalactan-responsive gut microbes. (A) Single-cell analysis of microbial metabolic activity in response to arabinogalactan. The percentage of deuterium incorporation (%CD) was measured in individual cells following 6 h of incubation with D_2_O, with or without arabinogalactan. The dots indicate single-cell measurements collected from each ten donors, while boxplots illustrate the spread of %CD values across conditions. Statistical testing across all donors revealed a significant increase in microbial metabolic activity following arabinogalactan treatment (ANOVA, *P* < 0.001, *n* = 607); (B) Phylogenetic and physiological profiling of representative arabinogalactan-responsive isolates. A mid-point rooted maximum likelihood phylogenetic tree was generated from near full-length 16S rRNA gene sequences of 16 representative strains isolated via RACS. Branch support values are based on 1,000 ultrafast bootstrap replicates. The tree generation was performed using the aligned sequences, with subsequent visualization annotated in iTOL^[51]^. The heatmap displays growth boost, calculated as the area under the growth curve in arabinogalactan-supplemented media relative to no-amendment control, based on three technical replicates per condition. Asterisks indicate statistically significant differences in AUC between the two conditions (Student’s *t*-test, *P* < 0.05, *n* = 6). Color. Bar plots show L2FC in abundance of each strain after 24 h of coculture with B. longum, as quantified by qPCR based on the average of three technical replicates. Asterisks (*) indicate significant differences in growth (Student’s *t*-test, *P* < 0.05, *n* = 6). ANOVA: Analysis of variance; RACS: Raman-activated cell sorting; iTOL: interactive tree of life; AUC: area under the curve; L2FC: log2 fold change; qPCR: quantitative PCR.

To identify metabolically active gut microbes responding to arabinogalactan, we performed D_2_O labeling followed by single-cell Raman microspectroscopy. Deuterium incorporation, quantified as the percentage of C–D bonds relative to total C–H and C–D (%CD), was used to assess microbial metabolic activity^[35]^. After 6 h of incubation, cells from arabinogalactan-supplemented microcosms showed significantly higher %CD values compared to their matched NA controls, indicating that arabinogalactan stimulated microbial metabolism across nearly all donors [Figure 2A]. A similar pattern of increased metabolic activity was also observed after 24 h of incubation [Supplementary Figure 6], confirming that the effect of arabinogalactan was consistent over time.

To isolate metabolically active taxa responsive to arabinogalactan, we next employed RACS following 6 h of incubation with D_2_O and arabinogalactan. A total of 98 strains were recovered from RACS-enriched fractions across all donors and classified via near-full-length 16S rRNA gene sequencing as *Bifidobacterium longum* (*n* = 45), *Collinsella aerofaciens* (*n* = 26), *Alistipes shahii* (*n* = 5), *Alistipes senegalensis* (*n* = 1), *Alistipes putredinis* (*n* = 2), *Alistipes onderdonkii* (*n* = 3), *Bacteroides uniformis* (*n* = 2), *Bacteroides stercoris* (*n* = 1), *Catenibacterium mitsuokai* (*n* = 6), *Dysosmobacter welbionis* (*n* = 1), *Eggerthella lenta* (*n* = 1), *Faecalibacterium prausnitzii* (*n* = 1), *Parabacteroides merdae* (*n* = 1), *Phascolarctobacterium faecium* (*n* = 1), *Phocaeicola coprocola* (*n* = 1), and *Ruminococcus bicirculans* (*n* = 1). These strains belonged to three different phyla: *Actinomycetota* (72 strains), *Bacteroidota* (16 strains), and *Bacillota* (10 strains) [Supplementary Figure 7 and Supplementary Data 7].

A total of 16 strains were selected as representative isolates for downstream analysis [Figure 2B]. One representative per species was chosen to assess its physiological response to arabinogalactan. To functionally characterize arabinogalactan responders, we selected 16 representative isolates from the 98 strains recovered by RACS, ensuring broad phylogenetic coverage while minimizing redundancy among closely related strains. A single *B. longum* strain was chosen for coculture assays based on its high recovery frequency (*n* = 45) and robust growth on arabinogalactan. Growth assays and coculture experiments were conducted in triplicate. Quantitative growth boosts were calculated as area under the curve (AUC) in arabinogalactan-supplemented versus no-amendment media, with statistical significance determined by Student’s *t*-test (*P* < 0.05). The log_2_ fold changes in coculture abundance, quantified by qPCR, represent mean ± SD from three replicates. Error bars and individual data points are included to illustrate replicate variation. The addition of arabinogalactan to the cultivation medium stimulated the growth of *B. longum*, *F. prausnitzii*, *C. mitsuokai*, *B. uniformis*, and *B. stercoris*.

Since the remaining strains had incorporated deuterium from D_2_O (indicating metabolic activity) but were unable to utilize arabinogalactan as a sole carbon source, we hypothesized that their growth might be indirectly supported by primary arabinogalactan degraders. *B. longum* was selected as the focal degrader as it was the most frequently isolated species and exhibited robust growth on arabinogalactan. To test this hypothesis, we performed coculture experiments in which each non-arabinogalactan-utilizing isolate was incubated with *B. longum* for 24 h in the presence of arabinogalactan. As expected, none of these strains grew in monoculture, but all displayed significant growth when cocultured with *B. longum*, suggesting that *B. longum* facilitated their proliferation through metabolic cross-feeding. Notably, the growth of *B. longum* itself was significantly reduced in cocultures with *Eggerthella lenta*, *Dysosmobacter welbionis*, *Ruminococcus bicirculans*, *Phascolarctobacterium faecium*, and *Phocaeicola coprocola*.

## DISCUSSION

Dietary fibers that selectively stimulate specific gut microbes are increasingly recognized for their potential to promote host health by modulating immunity, improving gut function, and contributing to the production of bioactive metabolites such as SCFAs^[7,13,52,53]^. Prebiotics such as inulin, FOS, and GOS have been explored for their capacity to selectively enrich microbial groups involved in carbohydrate fermentation and SCFA production^[8,38]^. These include *Bifidobacterium*, *Lactobacillus*, and *Faecalibacterium*, which are frequently associated with gut health and beneficial physiological functions^[38,54-57]^. However, their effects are not always consistent across studies, and the underlying microbial mechanisms are still not fully understood^[58,59]^. *Bifidobacterium*, in particular, is widely recognized as a keystone member of the gut microbiota with a central role in fiber degradation and ecosystem function^[60]^.

In this study, we evaluated the selective effects of arabinogalactan, a complex polysaccharide composed of the monosaccharides galactose and arabinose. Arabinogalactan consistently enriched both *Bifidobacterium* and *Gemmiger* across the donors at both the 6 and 24 h incubation time points. However, this pattern was not observed in unamended controls. Interestingly, galactose alone led to a consistent enrichment of *Bifidobacterium*, while arabinose selectively enriched *Gemmiger* at 6 h and *Blautia* and *Anaerostipes* at 24 h. These findings show that while each monosaccharide selectively stimulated specific taxa, the intact polysaccharide elicited a more coordinated response, simultaneously promoting both *Bifidobacterium* and *Gemmiger* in a way that was not observed with the individual sugars. Notably, similar enrichment patterns in response to galactose, arabinose, and arabinogalactan have been reported in previous studies and are consistent with our findings^[60-65]^. For instance, the enrichment of *Bifidobacterium* in response to galactose has been reported in transcriptomic studies examining growth on substrates rich in GOS and galactose^[61]^. In another study, *Blautia* proliferation increased via cross-feeding, following the release of arabinose from arabinoglycan-containing diets in the gut of malnourished mice^[60]^. Additionally, *B. longum* was found to be stimulated during the *in vitro* fermentation of arabinogalactan using a dynamic colon model^[65]^. These overlaps highlight the consistency of certain microbial responses, while our use of RACS-based isolation and strain-level functional validation provides new insights into active degraders and cooperative interactions, distinguishing our study from previous work.

*B. longum* was identified as a key species in arabinogalactan degradation, both in terms of abundance and metabolic function. It was the most frequently recovered taxon using RACS and demonstrated the ability to degrade arabinogalactan as a sole carbon source. Additional strains such as *F. prausnitzii*, *C. mitsuokai*, *B. uniformis*, and *B. stercoris* also showed the capacity to utilize arabinogalactan in monoculture. Among the additional responders, *F. prausnitzii* is notable for its well-known butyrate production, anti-inflammatory properties, and role in strengthening gut barrier function and modulating host immunity^[66]^. In contrast, several other isolates including *E. lenta*, *C. aerofaciens*, *P. coprocola*, and *R. bicirculans* were metabolically active but unable to grow on arabinogalactan alone, suggesting a dependence on external metabolic support. While previous studies have suggested that certain taxa such as *Bifidobacterium*, *Faecalibacterium*, and *Phascolarctobacterium* may contribute to arabinogalactan degradation^[18,67]^, our application of RACS enabled the targeted recovery of a wider set of responsive strains, expanding the current understanding of bacteria capable of utilizing arabinogalactan. Several strains that failed to grow on arabinogalactan in monoculture showed increased growth when cocultured with *B. longum*. These coculture dynamics point to *B. longum* acting as a keystone degrader that enables the growth of other taxa, potentially through metabolic byproducts or cross-feeding interactions^[68,69]^. In addition to its ecological role as a keystone degrader, *B. longum* is widely recognized for its beneficial impact on host health^[70]^. Numerous studies have shown that it contributes to intestinal barrier integrity, modulates immune responses, and inhibits pathogen colonization through the production of organic acids and competitive exclusion mechanisms^[71]^. The stimulation of *B. longum* suggests that arabinogalactan may offer reproducible bifidogenic benefits, reinforcing its potential as a functionally selective prebiotic.

Similar to *Bifidobacterium*, *Gemmiger* displayed a robust increase across the donor samples following arabinogalactan supplementation. However, despite its consistent enrichment, *Gemmiger* was not among the metabolically active strains isolated using RACS. This may be due to cultivation challenges or limitations in cell sorting, possibly related to its strict growth requirements^[72-74]^. While RACS is highly effective for detecting and isolating metabolically active cells, the technique has certain limitations. The laser used for Raman spectroscopy can transfer energy that may damage fragile cells and reduce their viability. Additionally, post-sorting recovery can be challenging for strictly anaerobic taxa that are highly sensitive to oxygen or require very specific growth conditions, which can limit successful cultivation after sorting. Interestingly, we successfully isolated *F. prausnitzii*, an oxygen-sensitive butyrate producer closely related to *Gemmiger*^[75]^. A 16S rRNA sequence comparison revealed that the dominant *Gemmiger* ASV shares 92.79% identity with *Faecalibacterium*, suggesting phylogenetic proximity and possibly overlapping ecological roles^[76,77]^. This suggests that both taxa may participate in functions such as arabinogalactan fermentation and butyrate production under anaerobic conditions. However, individual strains can differ in which sugars they break down, which enzymes they produce, and how well they grow under gut conditions. Therefore, detailed genome sequencing and experimental validation of substrate utilization and enzyme expression will be important to determine whether *Gemmiger* functionally overlaps with *F. prausnitzii* or contributes unique metabolic activities within the gut community.

Our findings highlight arabinogalactan as a functionally selective prebiotic that enriches specific gut microbes, most notably *B. longum*. Unlike other confirmed prebiotics such as inulin or GOS, which stimulate multiple primary degraders^[8,38,78]^, arabinogalactan targets a narrower group of responsive taxa. *B. longum* not only degrades the polysaccharide but also facilitates the growth of non-degraders, likely through cooperative metabolic interactions. These findings support the use of arabinogalactan as a candidate for targeted modulation of the gut microbiota. Unlike broad-spectrum prebiotics such as inulin, which are utilized by a wide range of gut microbes, arabinogalactan shows a narrower specificity, primarily stimulating *B. longum* and a few other taxa. This targeted effect may enable more precise modulation of microbiota composition. *Bifidobacterium* species are well known for efficiently fermenting short-chain oligosaccharides such as FOS and GOS using specialized glycoside hydrolases (GHs)^[14]^. Enzymes belonging to the families GH32, GH43 and GH68 cleave β-fructosidic linkages in FOS, while GH2, GH42 and GH43 families target galactosidic bonds in GOS^[79,80]^. β-Galactanases, including enzymes from the GH30, GH40, and GH43 families, are key for degrading the galactan backbone of arabinogalactan (AG), releasing galactose and short-chain GOS^[14]^. Although we did not directly investigate the genetic mechanisms underlying *B. longum*’s polysaccharide utilization, prior studies have reported multiple glycoside hydrolases in *B. longum* species, including GH30, GH40, GH43, GH51, GH121, GH146, and GH127 families, which are implicated in hydrolyzing arabinogalactan linkages^[67,81-83]^. Future work should include genome mining and transcriptomic profiling of *B. longum* grown on arabinogalactan to identify upregulated hydrolases and transport systems involved in its metabolism. Additionally, confirming whether similar GHs occur in other relevant taxa, including *Gemmiger* and the representative isolates identified in this study, will help clarify whether these organisms contribute to direct polysaccharide breakdown or rely primarily on cross-fed metabolites. Notably, publicly available genomes from *Gemmiger* spp. encode various GHs that are crucial for carbohydrate metabolism. Among these, the GH101 and GH112 families are of particular interest for their roles in the degradation of complex carbohydrates^[84]^. Likewise, *F. prausnitzii* genomes contain many different CAZymes, including GH43, GH33, GH78, GH4, and GH170. *F. prausnitzii* has also been reported to degrade rhamnose, which can be found as a side chain in plant polysaccharides such as arabinogalactan^[67,85]^. These findings support the hypothesis that taxa beyond *Bifidobacterium* may play a direct role in arabinogalactan breakdown. Future genome-resolved and transcriptomic analyses will be essential to validate the functional roles of these organisms and deepen our understanding of community-level carbohydrate utilization dynamics.

While 16S rRNA gene sequencing provided valuable insights into community-level shifts in response to arabinogalactan, it has notable limitations in taxonomic resolution, functional interpretation, and differential abundance testing. For instance, the 16S rRNA gene cannot reliably distinguish closely related species or infer the enzymatic capabilities responsible for fiber degradation. To address these challenges, we used RACS to isolate and validate metabolically active taxa at the strain level. This dual approach helped bridge the gap between community profiling and functional activity. However, future studies could benefit from incorporating multi-omics strategies such as shotgun metagenomics, metatranscriptomics, or metabolomics to resolve microbial interaction networks, uncover the genetic basis of arabinogalactan metabolism, and characterize host-relevant metabolic outputs with greater phylogenetic and functional resolution. In future studies, using absolute microbiome quantification approaches such as those based on internal standards or spike-ins could provide more accurate estimates of microbial abundance. Future work should clarify the metabolic pathways involved in arabinogalactan degradation, including the identity of breakdown products and their role in supporting non-degraders. Metabolite profiling of *B. longum* could reveal key compounds responsible for these effects. Additionally, profiling the metabolites produced by *B. longum* during this process may reveal key compounds that facilitate the proliferation of otherwise inactive taxa. Additionally, it will be important to investigate why the presence of certain strains in coculture reduces *B. longum* growth and how this influences overall carbon utilization dynamics. Quantifying how much arabinogalactan and its component sugars are consumed by both individual isolates and mixed microbial communities will provide clearer insights into their degradation capabilities and metabolic contributions. Finally, improving cultivation protocols and post-sorting recovery conditions may enhance the isolation of sensitive or slow-growing taxa, providing a more comprehensive view of the microbial networks shaped by selective prebiotics such as arabinogalactan.

There are several limitations to this study that should be acknowledged. First, our reliance on 16S rRNA gene sequencing restricts taxonomic resolution and does not provide direct information on the functional activities, metabolic pathways, or specific enzymes involved in arabinogalactan metabolism. However, this approach still allowed us to robustly identify key taxa that responded to arabinogalactan, providing valuable ecological insights. This limits our ability to draw conclusions about the metabolic mechanisms driving the observed microbial changes. In addition, we did not perform direct enzymatic assays, which would have helped confirm the involvement of particular enzymes in arabinogalactan degradation. All data are reported as relative abundances rather than absolute quantities. Implementing absolute quantification methods, such as digital PCR (dPCR) or spike-in standards, in future studies could enable more robust and quantitative assessments of microbial dynamics^[86]^. Despite this, the changes in relative abundance provided consistent and interpretable patterns across donors. The RACS technique, while powerful for isolating metabolically active cells, also presents challenges. Some taxa, such as *Gemmiger*, were not recovered, likely due to cultivation difficulties, sensitivity to laser exposure, or because strictly anaerobic organisms are more difficult to isolate with this method. As a result, some metabolically active and enriched taxa may have been missed among the isolates. Improved cultivation protocols and alternative single-cell approaches could help address this limitation. Furthermore, while coculture experiments indicated cross-feeding interactions, the precise metabolic intermediates exchanged between strains were not identified. Targeted metabolomics and comprehensive analysis of arabinogalactan utilization would be valuable for elucidating the nature of these metabolic exchanges and utilization patterns. Nonetheless, our experiments demonstrated clear ecological relationships and potential for cooperative interactions within the microbial community. Our study was conducted *in vitro* using fecal samples from healthy donors, but the effects of arabinogalactan on microbiota from individuals with dysbiosis or gut disease remain unexplored. Another limitation is the limited number of human donors included in this study. A larger number of donors and samples would make the results more reliable and improve our findings across more diverse populations. Future research involving clinical studies or animal models will be important to assess the impact of arabinogalactan supplementation under more physiologically relevant and disease-specific conditions. Finally, the absence of complementary multi-omics approaches, such as shotgun metagenomics, transcriptomics, or genome sequencing, limits the functional interpretation of our findings. Integrating these techniques in future research will be essential for a more comprehensive understanding of arabinogalactan’s impact on the gut microbiome. Despite these limitations, the integrative methods used in this study provide a strong foundation for future investigations.

In conclusion, this study demonstrates that arabinogalactan acts as a functionally selective dietary fiber that consistently enriches beneficial members of the gut microbiota, most notably *B. longum*. Using an integrated *ex vivo* framework combining 16S rRNA gene sequencing with RACS, we identified key taxa actively responding to arabinogalactan supplementation across microbiota from multiple human donors. Among the 98 metabolically active strains isolated, *B. longum* accounted for approximately 46% and was selected for detailed physiological characterization based on its high recovery rate and known role in fiber degradation. *B. longum* emerged as a central degrader, directly utilizing arabinogalactan and facilitating the growth of non-degrading strains through metabolic cross-feeding. While isolates from *Bacteroidota* and *Bacillota* were also recovered, this study focused on *Actinomycetota* due to the dominance of *Bifidobacterium* in the RACS-sorted fraction. The targeted recovery and physiological assessment of isolates from three dominant gut phyla *Actinomycetota*, *Bacteroidota*, and *Bacillota* revealed that most metabolically active strains belonged to *Actinomycetota*, primarily due to the high prevalence of *Bifidobacterium*. These results underscore arabinogalactan’s selective influence on gut microbial community composition and highlight the potential of single-cell techniques like RACS to uncover functionally important microbial interactions within complex ecosystems.

## DECLARATIONS

### Availability of data and materials

All 16S rRNA gene amplicon sequencing data generated during this study are available in the NCBI Short Read Archive under the accession number PRJNA1244259. The sequences of RACS-isolated strains following arabinogalactan supplementation have been submitted to GenBank under accession numbers PQ407681 to PQ407778. The Raman spectroscopy dataset has been submitted to the BioStudies database and is accessible through the MicrobioRaman platform under the accession number S-MBRS14.

### Copyright

© The Author(s) 2025.
