# Supplementary material for: Evaluating the prebiotic activity of arabinogalactan on the human gut microbiota using 16S rRNA gene sequencing and Raman-activated cell sorting
Source: Microbiome Res Rep. 2025 Aug 14;4(3):30. doi: 10.20517/mrr.2025.29 (PMC12540051; doi:10.20517/mrr.2025.29)
Supplement: Supplementary file 1 [file mrr-4-3-30-SupplementaryMaterials.pdf]

Supplementary Materials

Evaluating the prebiotic activity of arabinogalactan on the human gut microbiota using 16S rRNA gene sequencing and Raman-activated cell sorting

Hamid Rasoulimehrabani[1,2], Sanaz Khadem[1], Adnan Hodžić[1], Miriam Philipp[1], Rebecca Gallo[1,2], Georgi Nikolov[1], Joana Séneca[3], Julia Ramesmayer[3], Patrik Sivulič[4], David Berry[1,3]

[1]Center for Microbiology and Environmental Systems Science, Department of Microbiology and Ecosystem Science, University of Vienna, Vienna 1030, Austria.
[2]Doctoral School in Microbiology and Environmental Science, University of Vienna, Vienna 1030, Austria.
[3]Joint Microbiome Facility of the Medical University of Vienna and the University of Vienna, Vienna 1030, Austria.
[4]Department of Natural Drugs, Faculty of Pharmacy, Masaryk University in Brno, Brno 612 00, Czech Republic.

Correspondence to: Prof. David Berry, Centre for Microbiology and Environmental Systems Science, Department of Microbiology and Ecosystem Science, University of Vienna, University Biology Building (UBB), Djerassiplatz 1, Vienna 1030, Austria. E-mail: david.berry@univie.ac.at

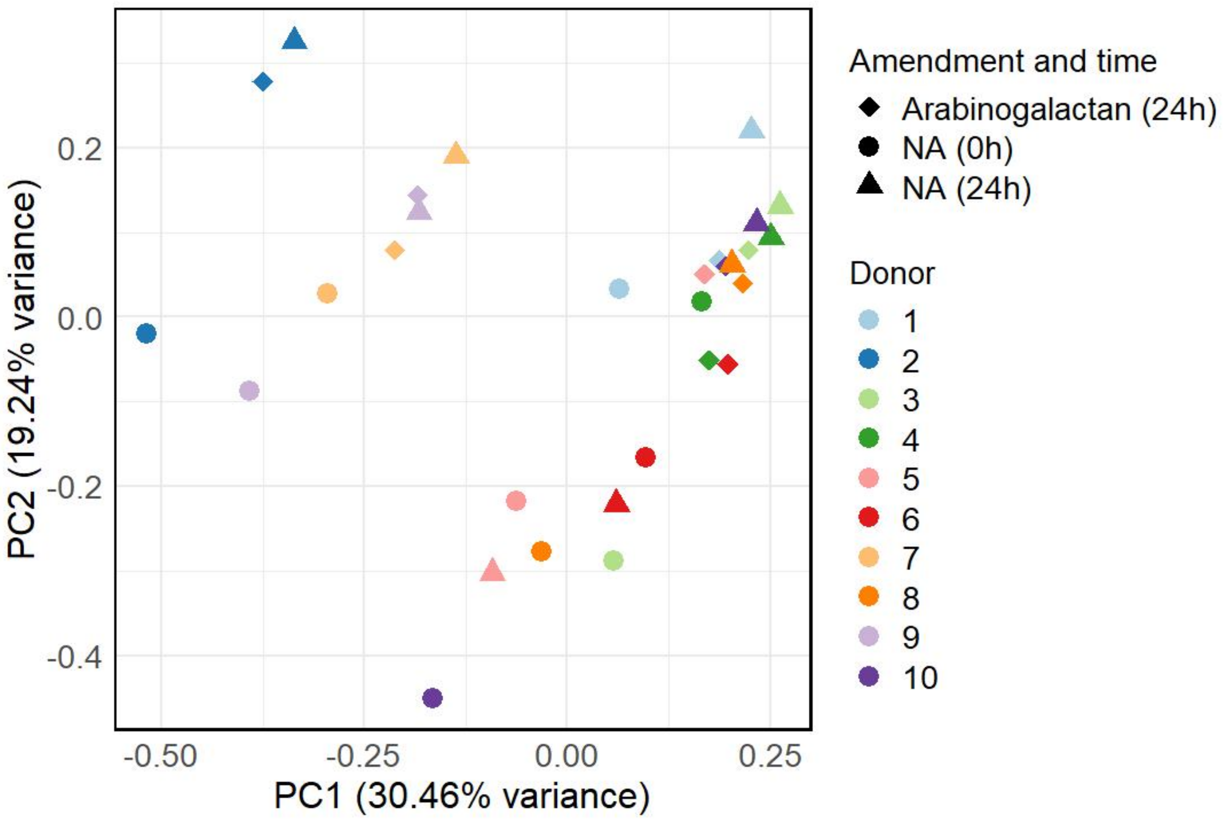

Supplementary Figure 1. Principal coordinate analysis (PCoA) ordination of genus-level microbiome profiles showing clustering of samples incubated with Arabinogalactan for 24 hours, compared to no amendment (NA) samples at 0 and 24 hours. Color and shape indicate donor identity and treatment condition, highlighting both individual and treatment-related differences.

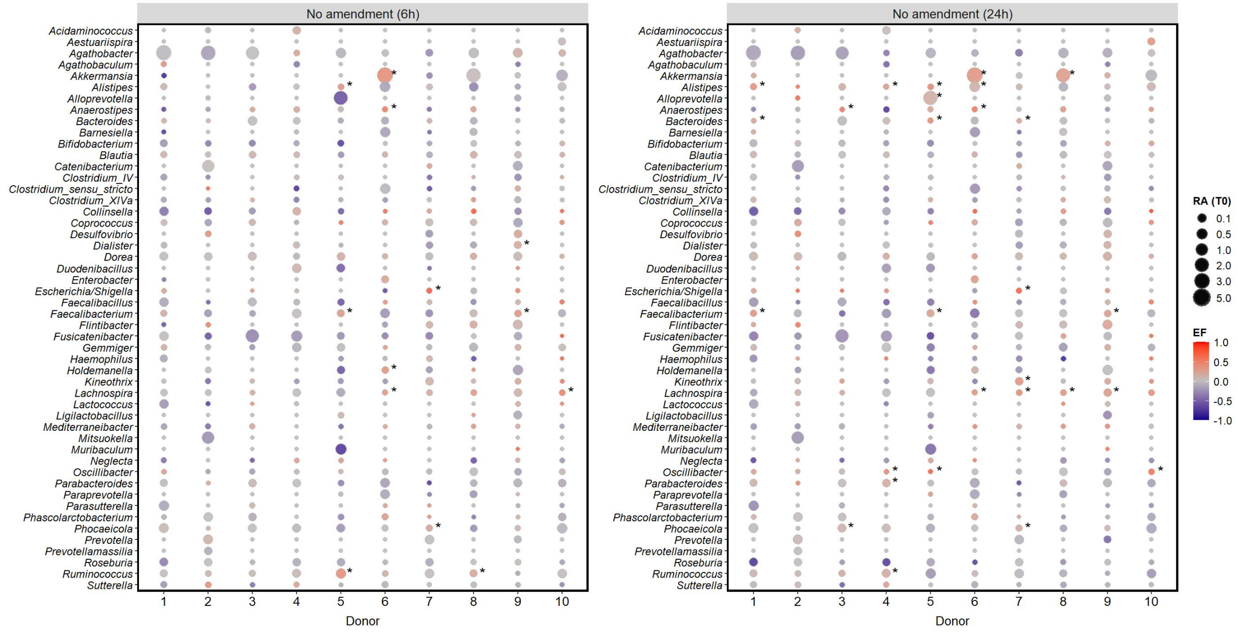

Supplementary Figure 2. No amendment controls (NA). Enrichment patterns of dominant bacterial genera after 6 and 24 h incubation in no amendment. Bubble size indicates the relative abundance at 0 h, and color indicates the scaled enrichment factor (EF), as described in the Materials and Methods section. Genera with significant enrichment in individual donors are indicated by an asterisk, while those consistently enriched across all donors are highlighted with a black outline (DESeq2, Wald Test p.adj < 0.05, n = 30 samples).

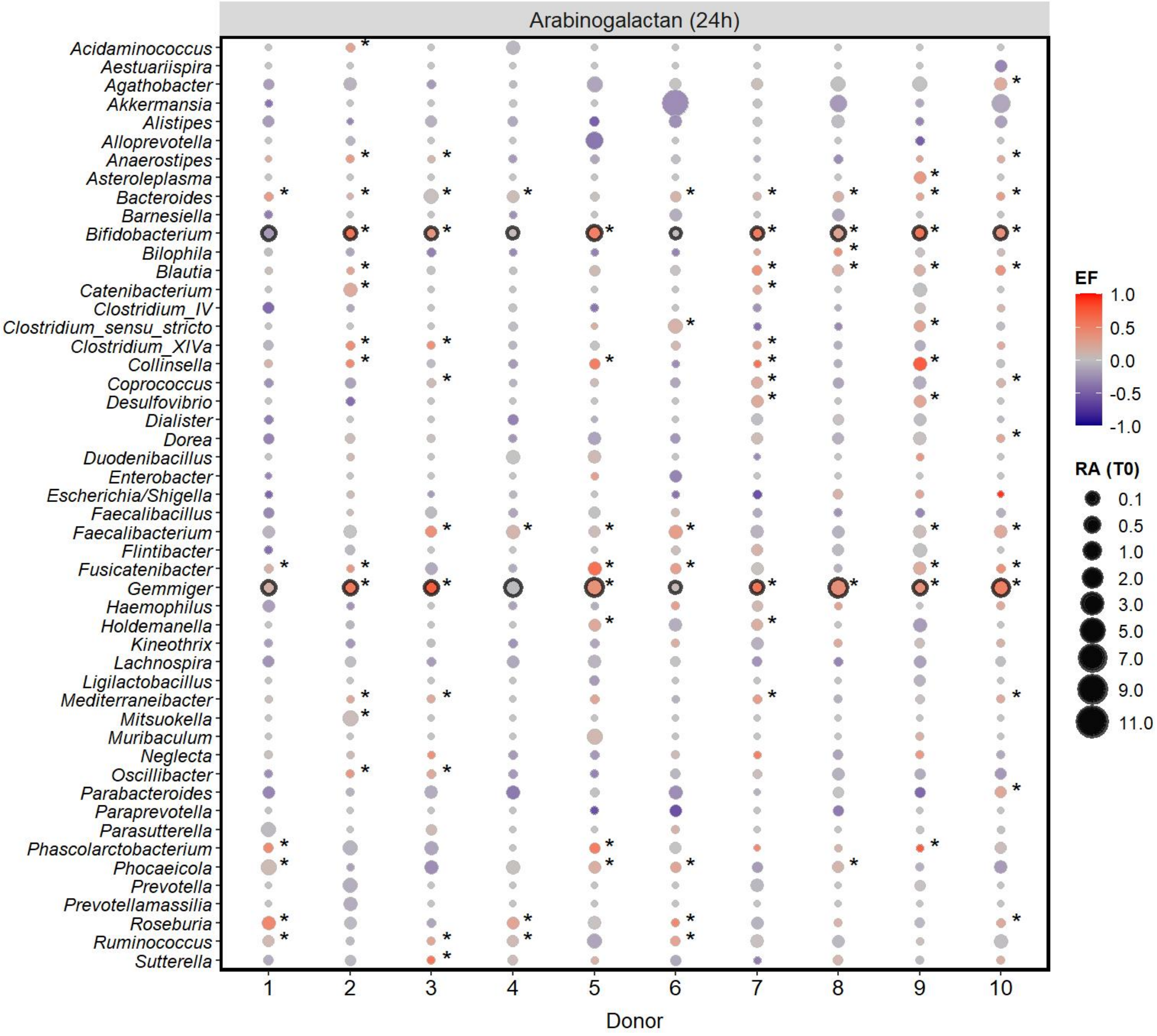

Supplementary Figure 3. Enrichment patterns of dominant bacterial genera after 24 h incubation with arabinogalactan. Bubble size indicates the relative abundance at 0 h, and color indicates the scaled enrichment factor (EF), as described in the Materials and Methods section. Genera with significant enrichment in individual donors are indicated by an asterisk, while those consistently enriched across all donors are highlighted with a black outline (DESeq2, Wald Test p.adj < 0.05, n = 30 samples).

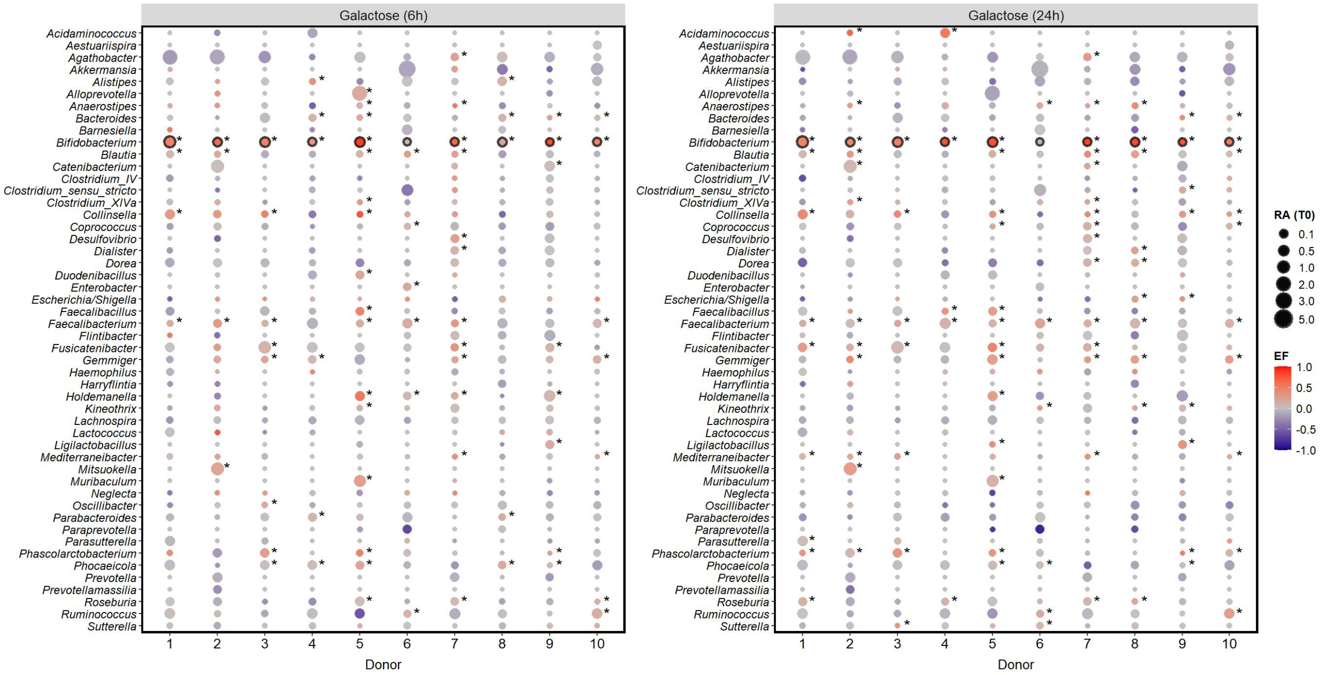

Supplementary Figure 4. Enrichment patterns of dominant bacterial genera after 6 and 24 h incubation with galactose. Bubble size indicates the relative abundance at 0 h, and color indicates the scaled enrichment factor (EF), as described in the Materials and Methods section. Genera with significant enrichment in individual donors are indicated by an asterisk, while those consistently enriched across all donors are highlighted with a black outline (DESeq2, Wald Test p.adj < 0.05, n = 30 samples).

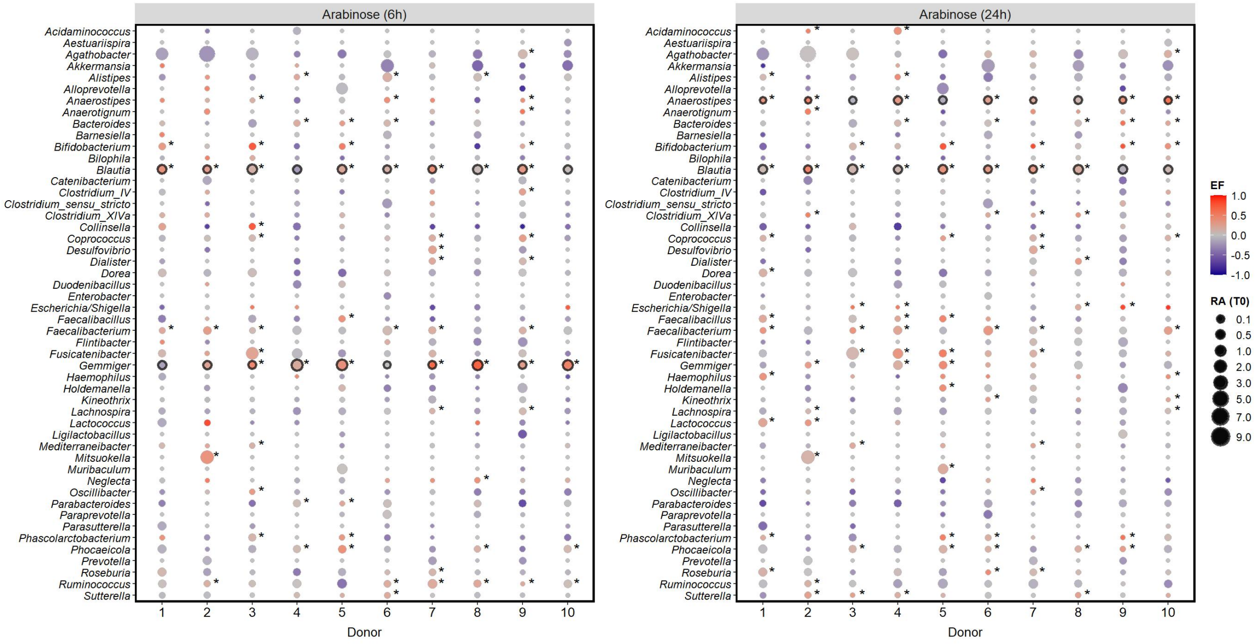

Supplementary Figure 5. Enrichment patterns of dominant bacterial genera after 6 and 24 h incubation with arabinose. Bubble size indicates the relative abundance at 0 h, and color indicates the scaled enrichment factor (EF), as described in the Materials and Methods section. Genera with significant enrichment in individual donors are indicated by an asterisk, while those consistently enriched across all donors are highlighted with a black outline (DESeq2, Wald Test p.adj < 0.05, n = 30 samples).

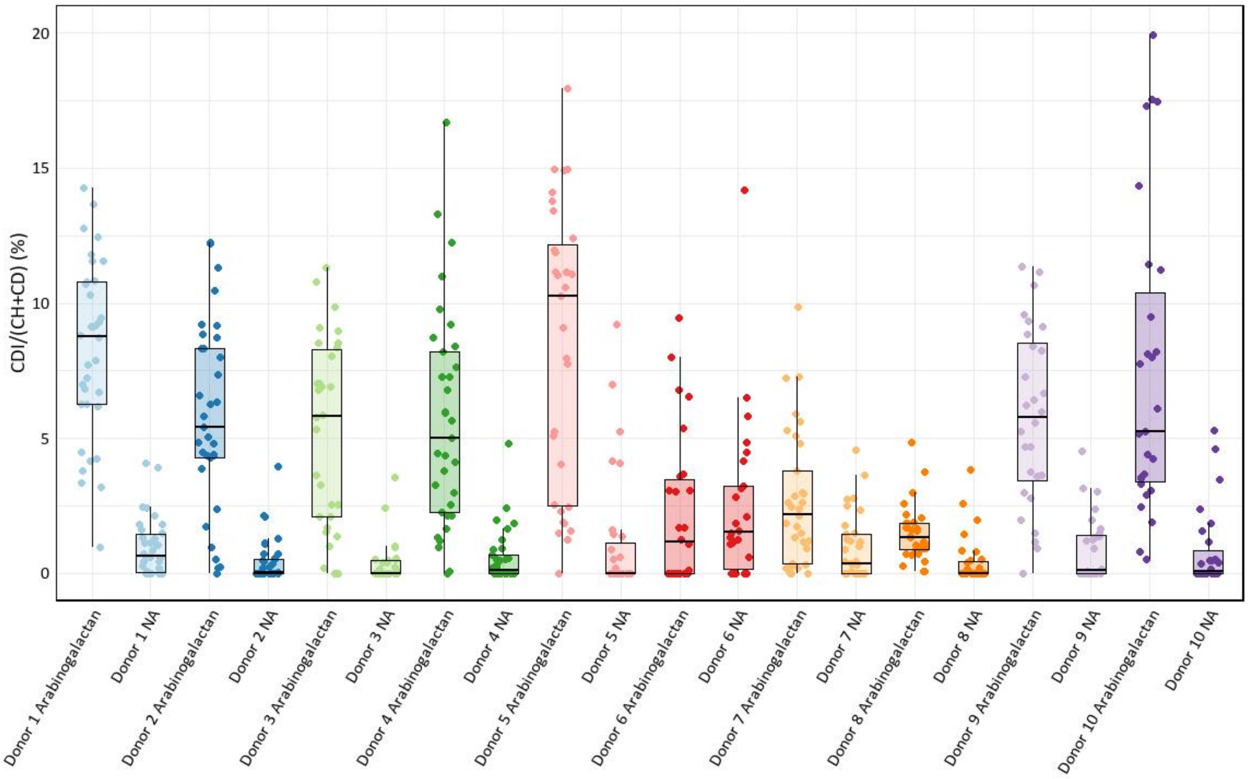

Supplementary Figure 6. Single-cell analysis of microbial metabolic activity in response to arabinogalactan. The percentage of deuterium incorporation (%CD) was measured in individual cells following 24 h incubation with D_2O, with or without arabinogalactan. The dots indicate single-cell measurements collected from each ten donors, while boxplots illustrate the spread of %CD values across conditions. Statistical testing across all donors revealed a significant increase in microbial metabolic activity following arabinogalactan treatment (ANOVA, $p<0.001$, $n=611$).

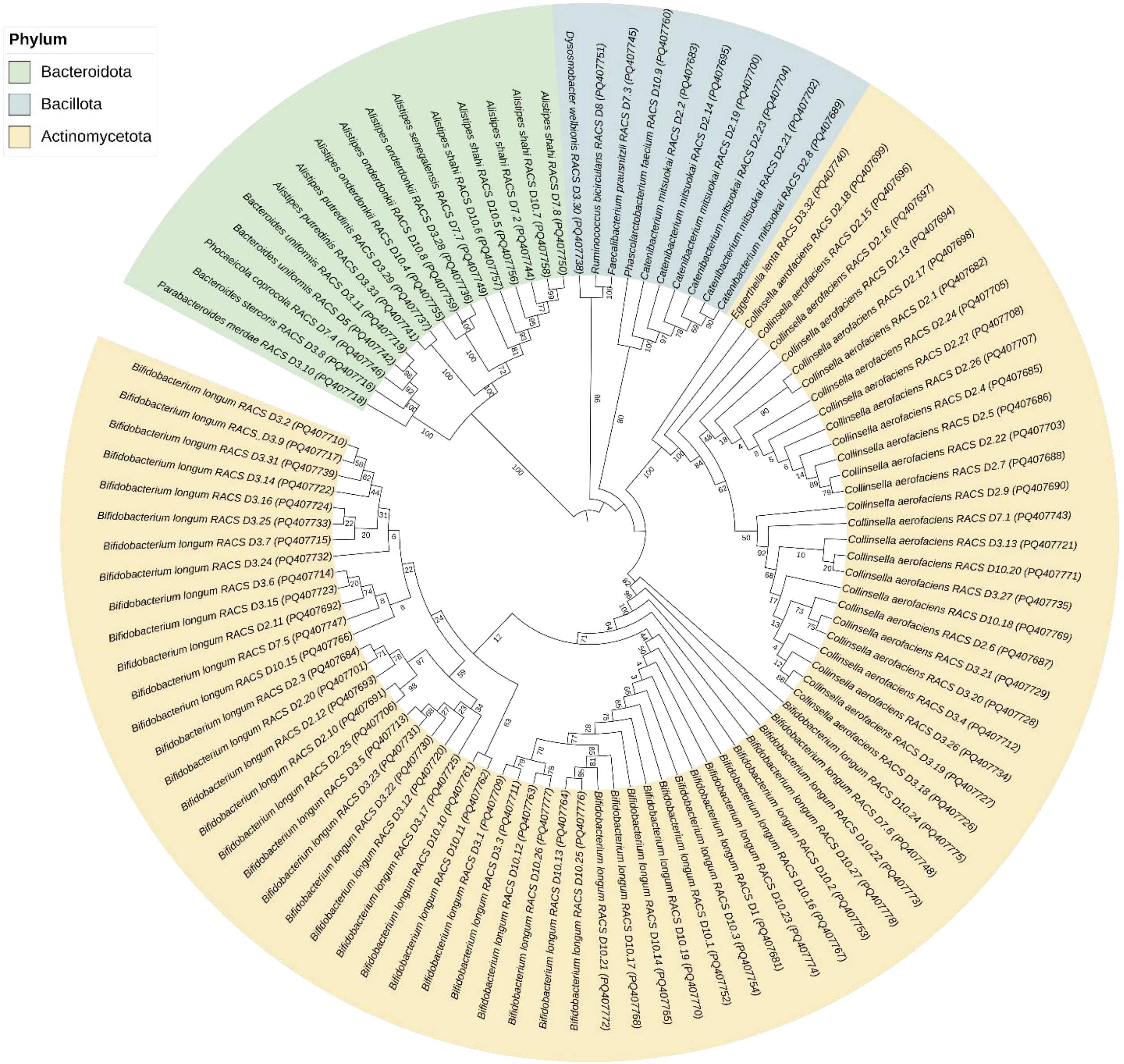

Supplementary Figure 7. Phylogenetic characterization of metabolically active cells sorted by RACS. Phylogenetic tree of 98 bacterial strains isolated using Raman-activated cell sorting (RACS) following 6 hours incubation with D_2O and Arabinogalactan. Isolates were classified based on near-full-length 16S rRNA gene sequencing and represent 16 species spanning three phyla: Actinomycetota (n = 72), Bacteroidota (n = 16), and Bacillota (n = 10). The phylogenetic tree was generated using the maximum likelihood algorithm in IQ-TREE, rooted at the midpoint, and branch support was calculated using 1000 ultrafast bootstrap replicates. Tree visualization and annotation were performed using iTOL, with phylum-level taxonomy indicated by branch color.

Supplementary Table 1. Number of D-labeled and unlabeled cells analyzed during RACS for each donor following AG supplementation

Donor	1	2	3	4	5	6	7	8	9	10
Total analyzed cells (n)	821	798	239	174	182	843	169	548	192	782
D-labeled cells (n)	5	28	63	7	60	25	26	40	24	27
Labeled (%)	0.6	3.5	26.35	4.02	32.96	2.96	15.38	7.29	12.5	3.45
Colonies recovered (n)	1	27	33	0	1	0	8	1	0	27
Recovery rate (%)	20	96.4	52.3	0	1.6	0	30.76	2.5	0	100

The total number of sorted cells and the number of colonies recovered are shown. The recovery rate indicates the percentage of colonies obtained from the sorted D-labeled cells.
